# The College of Nursing, Limited

**Published:** 1917-11-03

**Authors:** Vera Matheson

**Affiliations:** Secretary, Irish Board.


### THE MATRONS' AND SISTERS' DEPARTMENT

### THE COLLEGE OF NURSING, LIMITED.

### Its Scotch and Irish Branches.

**By Miss VERA MATHESON, B.A., Secretary, Irish Board.***

With regard to the Scotch and Irish Branches, their local governing bodies are Boards and they have proportionate representation on the College Council. Their Council members are elected by the Scottish and Irish sections of the nurses on the Register respectively. They manage their own affairs, keep their Branch Registers, deal with their own nurses, appoint their own examiners and officials, subject only to the formal confirmation of the Council; and in fact have their own way in everything so long as that way is in conformity with the declared objects of the College.

Who can be on the Register, and how does one get there? During the period of grace, that is, during the three years immediately following the foundation of the College, certain regulations have been laid down enabling fully-trained nurses to become members of the College without passing any further examination. They must have had three years' general training in a recognised hospital, or else two years' general training followed by two years' *bona-fide* nursing. For nurses who finished their training before January 1, 1900, special conditions have been made. All candidates apply on the given application form and send this application, together with a copy of their certificate of training, to the Secretary of the College in London or, if trained in Scotland or Ireland, to the Scotch and Irish Secretaries respectively. When their case has been considered the Council notifies them of their acceptance if they have been accepted, and they then send the registration fee of one guinea, and receive in return the certificate of membership and registration. When the period of grace is over, all candidates will have to have the training and pass the examination prescribed by the Council.

It has been objected that the College of Nursing is a lay body, that—to quote the published manifesto of the Irish Nursing Board—"amateurs are out to capture the control of our profession, and subject the professional woman to the ignorant rule of the aristocrat and the plutocrat." It is quite true that the Chairman of the College of Nursing is neither a doctor nor a nurse. It is also true that on the present Council of the College there are two laymen. One is Mr. Andrew Beattie, the hon. treasurer of the Irish Board; the other is Mr. Minet, a lawyer and the chairman of the Nightingale Fund, whose wife is a trained nurse. All the other members of the Council are doctors or nurses. And in actual fact the control of the College by laymen is an impossibility, for the simple reason that two-thirds of the Council will always be elected by the nurses on the Register: two-thirds is a big majority and means control. The nurses therefore have the control, and if they do not use it, why, then, it is their own fault.

#### Where Ireland comes in.

Now, where does Ireland come in in all this? Well, as a matter of fact poor Ireland nearly got left in the lurch and not included in the scope of the College at all. Fortunately, however, there were many Irish doctors and nurses who saw what a calamity that would be, and they set to work to form an Irish Branch of the College. This Irish Branch was formed in February of this year, and it manages the affairs of the College in Ireland. Ireland has her own peculiar conditions in nursing as in everything else, and it takes an Irishman or an Irishwoman to understand them. But that's no reason why we Irish nurses should get left out of a big scheme like the College, and remain in isolation from the rest of the English-speaking nursing world. We have a splendid chance now, and if we'll only wake up and keep awake we can share all the benefits of the College, and at the same time be free to develop along our own lines. It has been said, "Why have an altogether independent body? Why have anything to do with an English College?" I think that can be explained. A large proportion of Irish-trained nurses take up work in England. They will always do so. No matter what happens to Ireland politically, nurses will always want to look for work in England. Even when Ireland is entirely separated from and independent of England, even if Sinn Fein becomes the government of the country and Irish the spoken language of the nation, our nurses will still want to go and work in England and the Colonies. And, therefore, they want to be on the same footing as English nurses. And the most straightforward way of being on the same footing is to hold the same certificate and be on the same Register, and have the same professional advantages as English nurses. Of course, if Ireland had wakened up *before* the College came, and had *then* started an organisation of her own with the same objects, it would perhaps have been superfluous to have had an Irish Branch of the College. But there was no such organisation. The Irish Branch of the College of Nursing was the first to tackle the nursing problem in this country. Another organisation has since arisen, which, disapproving of the scope and constitution of the College, proposes to undertake a somewhat similar work in its own way. In so far as it is a sign of life and movement, the Irish Nursing Board is good. In so far as it is causing division and thereby preventing progress, it is *bad*. There are several very knotty problems to be tackled in the endeavour to bring about reform in Ireland, and an enormous amount of prejudice to be overcome. If we are to tackle these problems successfully, we Irish nurses must unite. And to

**COLLEGE OF NURSING, LIMITED**—(*continued*).

the nurses present I do most strongly appeal. Why will you degrade a noble profession by allowing political considerations to interfere or to bias you? You know that politics, division, and disunion have been the ruin of Ireland.

No Politics; Goal a Well-organised Profession.

What has nursing to do with these things? It has no political traditions to make cleavage necessary, or to prevent a natural union of all nurses. Its ministrations have always been extended alike to rich and poor, to native and foreigner, to Christian, Jew, Mohammedan, Buddhist, and atheist. You would not refuse to nurse your worst enemy if he needed it. Your nursing instinct would be too strong for you. Why, then, soil the purity of such an instinct by allowing any other considerations but those of your profession to bias your outlook upon its future? Our love for every stick and stone of Ireland, even our conviction that everything Irish is superior to everything English, should make us all the more eager to be proud of our Irish nursing profession and to do our utmost for it. We have our good points as nurses, and we have our bad ones. Setting out from the same starting-point as English nurses, running the same course to the same goal, we shall keep our sense of proportion, overcome our defects, develop our good qualities, and by co-operation and good-fellowship more surely prove the excellence of Irish nurses and Irish training, than if we cut ourselves off into an isolated partition. The partition can, perhaps, be made very beautiful, but we soon tire of the most beautiful things if we are compelled to look at them always and never see anything else. We must make our own corner perfect, but at the same time we must knock down the enclosing walls, and so be able to see the view beyond. Let us present a united front to the problems that face us. If all we Irish nurses join the College of Nursing now we shall be in a splendid position, able to govern our own profession on the broadest lines, to get all we want for Irish nurses, to take advantage of every advance in science and in the progress of human thought. If we can only unite, we shall find a method of solving every problem, no matter how difficult, whereas if we allow a difference on some unimportant point to grow and grow and obscure the end we have in view, we shall fail to accomplish our purpose: a tiny bit here and a tiny bit there we may achieve scattered arcs of the circle, but never the perfect whole. Let us sweep away the cobwebs which stretch themselves across our view, and see clearly the goal and the path to that goal. The goal is a thoroughly well-organised nursing profession, formed of a body of fully educated, fully trained women, with high ideals, accurate knowledge, and skilled touch, co-operating in the wise management of their profession and the perpetuation of its highest ideals and standards. The path to that goal is an organisation which will make possible all these things, and which, by embracing the whole of the profession, will enable nurses to be fully trained, fully educated, and adequately paid, and will give to every nurse the opportunities to work along the line of her profession which most appeals to her. By thus developing the best that is in every nurse, and enabling her to give that best where it is needed, all will perform the great service for which nurses exist, the alleviation of the suffering of humanity.

---

*At a representative meeting of nurses held on October 29, 1917, at the Royal College of Physicians, Dublin, Miss Vera Matheson, B.A., Secretary to the Irish Board of the College of Nursing, Limited, delivered an able and interesting address, from which the above is taken. Dr. George Peacocke presided, and there were also present Sir Andrew Horne, M.D., and Sir John Moore, M.D. Some interesting questions were asked and answered, but there was no discussion.

